# Conjugated topological interface-states in coupled ring resonators

**DOI:** 10.1038/s41598-021-91288-y

**Published:** 2021-06-08

**Authors:** Yu-Chuan Lin, Bo-Yu Chen, Wen-Jeng Hsueh

**Affiliations:** 1 Photonics Group, Department of Engineering Science and Ocean Engineering, National Taiwan University, 1, Sec. 4, Roosevelt Road, Taipei, 10660 Taiwan; 2 Taiwan Instrument Research Institute, National Applied Research Laboratories, 20, R&D Rd. VI, Hsinchu Science Park, Hsinchu, 30076 Taiwan

**Keywords:** Microresonators, Integrated optics, Optoelectronic devices and components

## Abstract

The optical properties of topological photonics have attracted much interest recently because its potential applications for robust unidirectional transmission that are immune to scattering at disorder. However, researches on topological series coupled ring resonators (T-SCRR) have been much less discussed. The existence of topological interface-states (TIS) in the T-SCRR is described for the first time in this article. An approach has been developed to achieve this goal via the band structure of dielectric binary ring resonators and the Zak phase of each bandgap. It is found that an ultra-high-Q with complete transmission is obtained by the conjugated topological series coupled ring resonators due to the excitation of conjugated topological interface-states, which is different from those in conventional TIS. Furthermore, the problem of transmission decreases resulting from high-Q increases in the traditional photonic system is significantly improved by this approach. These findings could pave a novel path for developing advanced high-Q filters, optical sensors, switches, resonators, communications and quantum information processors.

## Introduction

Nowadays, topological interface-states (TIS) in photonic structures have been developed into a very useful physical effect with numerous applications. TIS have the extraordinary property of robust one-way transportation against perturbations and defects, which have been a topic of great interest. The field of topological photonics is dealing with this phenomenon. TIS have been obtained in various photonic crystals, superlattices and hetero-structures^1–8^, which show the extraordinary properties at their boundaries or interfaces^9–11^. They have inspired recent studies on the topological structures of optics and photonics field. One of the main purposes of these studies is to realize advanced photonic devices that are not sensitive to manufacturing defects, such as topological hybrid silicon micro-lasers^12^, topological photonic crystal nano-cavity lasers^13^, tunable valley topological photonic crystals^14^ and room-temperature lasing from nano-photonic topological cavities were studied^15^. A photonic Floquet topological insulator in a fractal lattice was proposed and robust transport along the outer and inner edges of the fractal landscape was demonstrated^16^.

The use of one-dimensional conjugated topological photonic crystals (CTPC) to achieve robust high-Q and complete transmission was reported in our previous research^17^. Series coupled ring resonators (SCRR) are one of the most promising applications in integrated all-optical chips and quantum photonic chips owing to their extraordinary optical properties. However, studies on the use of TIS to obtain robust transportation against impurities and defects by the SCRR have been much less discussed. Compared to other kinds of traditional optical resonators, such as Fabry-Perot cavities^18,19^ and photonic crystal cavities^20–22^, the SCRR do not need strong refractive index discontinuities along the propagation direction of light, while reducing scattering and the effect of losses^23–25^. However, these properties make their performance highly susceptible to variations in manufacturing and to fluctuations in impurities. Therefore, an interesting topic has emerged regarding whether it is possible to find a way to overcome the aforementioned problems. Whether TIS can also be excited in the SCRR is also a very interesting subject. Only a few initial studies are available currently, such as topological phases and the bulk-edge correspondence in 2D photonic quantum well rings^26^. In addition, an active Su-Schrieffer-Heeger (SSH) photonic structure has been fabricated on InGasP quantum wells and the lasing TIS properties in an SSH photonic quantum well ring arrays have been investigated^27^. The physical and practical applications of topological phenomena in the topological series coupled ring resonators (T-SCRR) have not been shown so far. It is worth investigating whether the T-SCRR exhibits the same topological phenomenon as traditional topological photonic crystals.

In order to answer these questions, we introduced the concept of topological protection into the T-SCRR and discuss how these extraordinary optical properties of CTIS in the conjugated topological series coupled ring resonators (CT-SCRR) lead to the application of new photonic devices. The study results show that ultra-high-Q resonance and perfect transmission are obtained by the CT-SCRR due to excitation of CTIS. The problem of transmission decreases resulting from high-Q increases in the traditional photonic crystals is significantly improved by this approach. These high-Q resonances of topological interface-states enable the photonic structures to be excellent filters, sensors and communications.

## Model and method

**Figure 1:**
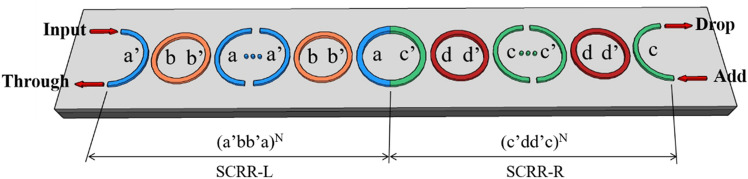
Schematic diagram of the T-SCRR consists of SCRR-L and SCRR-R.

A silicon-based SCRR was considered which consists of SCRR-L and SCRR-R as shown in Fig. 1. The SCRR-L consists of N-th half micro-rings denoted (a′bb′a)^N^, and the SCRR-R consists of N-th half mico-rings denoted (c′dd′c)^N^, respectively. The radius of ring a, b, c and d are denoted as R_a_, R_b_, R_c_ and R_d_. The length of the SCRR-L is denoted as D1, and the length of the SCRR-R is denoted as D_2_, where D_1_ = R_a_ + R_b_, D_2_ = R_c_ + R_d_. The normalized frequency is denoted as Ω = ωD/2πc, where c is the speed of light in a vacuum. The period number and the filling factor of structure are denoted as N and FS, where FS_L_ = R_a_ / R_a_ + R_b_, FS_R_ = R_c_ / R_c_ + R_d_. The variation in the value of FS_L_ and FS_R_ represent change in the ring dimension. If the sum of two filling factor equal to 1, such as FS_L_ = 0.25 and FS_R_ = 0.75, the two SCRR are referred to as conjugated. The coupling coefficient between ring a and b is denoted as C_ab_. The coupling coefficient between ring c and d is denoted as C_cd_. The coupling coefficient has a better coupling effect between 0.1 and 0.3 according to previous reports^28^. The transfer matrix method is utilized to calculate the behavior of optical transmission, and the high quality factor is considered at different period numbers, coupling coefficient and filling factor for the CT-SCRR. The detailed calculation and definition are shown in the supplementary information. Topological invariants play more and more important role in modern physics. The concept of topology has also been extended to diverse photonic system. The topological properties of the bulk band can be characterized by topological invariants, which are proportional to the Berry phase picked up by a particle moving across the first Brillouin zone. In two-dimensional system, the topological invariants is characterized by the Chern number, which is proportional to the Berry phase enclosing the Brillouin zone^1,29^. However, in the one-dimensional system, the topological invariants can be characterized by the Zak phase, a special kind of Berry phase defined for one-dimensional bulk band^30,31^. More specifically, the Zak phase refers to Berry’s phase picked up by a particle moving across the Brillouin zone, which characterizes the topological properties of Bloch band in a one-dimensional period system. Inspired by the above theories, we further investigated in diverse photonic system, such as series coupled ring resonators and photonic crystals. To further understand how we produce topological interface-states in this study, we provide an explain on the topological properties in a one-dimensional system. For a one-dimensional system with inherent mirror symmetry, the Zak phase of this system takes up a quantized value of 0 or π. The relation between the sign of reflection phase and the Zak phase is given by^7,32^:

1 $${\theta }_{n}^{Zak}={\int }_{BZ}i\left({\psi }_{k}\left|\nabla k\right|{\psi }_{k}\right)dk $$

where ψk is the normalized Bloch eigen-function of a state with wave vector k. BZ is denoted Brillouin zone. One-dimensional topological photonic crystals with inversion symmetry always have the Zak phase quantized at either 0 or π. In other words, for a 1D system with inherent mirror symmetry, the Zak phase of this system takes up a quantized value of 0 or π. The relation between the sign of reflection phase and the Zak phase is given by^32–34^:

2 $$\frac{\mathrm{sgn}\left({\varnothing }_{n+1}\right)}{\mathrm{sgn}\left({\varnothing }_{n}\right)}=-\mathrm{exp}\left(\mathrm{i}{\theta }_{n}^{Zak}\right),\mathrm{ with }\quad {\theta }_{n}^{Zak}=0 or \pi $$

where ∅_n_ is the reflection phase of the nth bandgap and ${\theta }_{n}^{Zak}$ is the Zak phase of the nth band, which is the band sandwiched by the (n + 1)th and nth bandgap.

## Results and discussions

Consider a topological series coupled ring resonator (T-SCRR), which consists of SCRR-L and SCRR-R as shown in Fig. 1. The SCRR-L consists of N-th half micro-rings denoted (a′bb′a)^N^, and the SCRR-R consists of N-th half mico-rings denoted (c′dd′c)^N^, respectively. In order to construct the TIS produced in this case, we show the topological phase transition of two micro-ring resonators. Topological phase transition plays an important role in the optical properties of topological interface-states, which is characterized by the Zak phase change and the reflection phase change. In other words, the topological interface-states can be excited when the Zak phase changes from 0 to π or from π to 0 and the reflection phase changes from positive to negative or from negative to positive. Moreover, the main difference between the TIS and the usual interface-states (IS) is the Zak phase change. The TIS must satisfy both the changes of reflection phases and Zak phases. The usual IS only satisfies the reflection phase change. This study finds that although the TIS and the usual IS can be excited in the micro-ring resonators, their optical properties are unusually different including the transmittance and the quality factor. These unique and interesting optical properties are discussed in more depth herein, and the difference optical properties between topological interface-states and usual interface-states in the SCRR are also compared.

**Figure 2:**
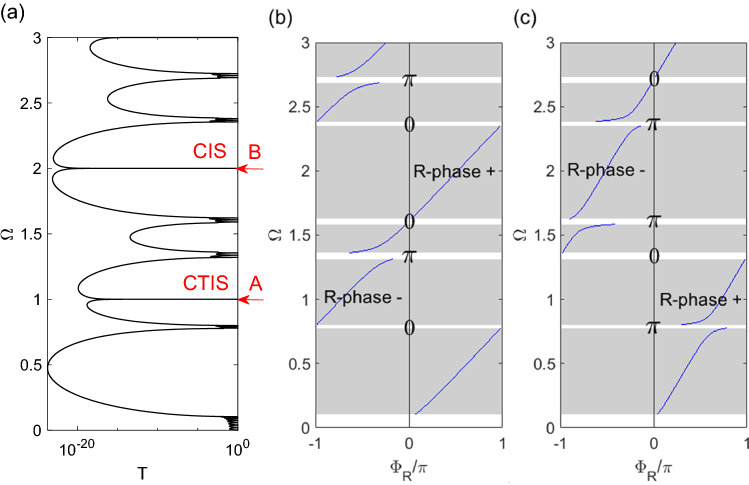
(**a**) Transmission spectrum of CT-SCRR, (**b**) Reflection phases of SCRR-L, (**c**) Reflection phases of SCRR-R. The parameters of the considered structure are FS_L_ = 0.37, FS_R_ = 0.63, R_a_ + R_b_ = 50 um, C_ab_ = 0.1, N = 4.

The topological interface-state is related to the topological phase transition of two SCRR in the band gap. The reflection phase changes and the Zak phase changes directly govern the topological properties. Analogous to Su-Schrieffer-Heeger (SSH) model in electronic systems^35,36^, topological interface-states emerge when two periodic SCRRs with different Zak phases are connected. The detailed calculation and definition are shown in the supplementary information. In this study, we show the reflection phases of two types of SCRR. We can observe from the transmission spectrum in Fig. 2a that CTIS is excited at the normalized frequency Ω = 1 and CIS is excited at the normalized frequency Ω = 2. The difference between these two states is the topological phase transition. The TIS indicated by the red arrow has the opposite reflection phase sign and also undergoes the Zak phase transition from π to 0 and 0 to π. However, the IS indicated by the red arrow only has the opposite sign reflection phase. Although these two excited states have the characteristics of complete transmissions, their optical properties are very different. This is a very interesting phenomenon that will be discussed later. We also observed that the TIS and the IS have different shapes in their transmission peaks. The transmission peak of TIS is asymmetric in shape but the transmission peak of IS is completely symmetric in shape. Figure 2b,c show the reflection phases changes and the Zak phase transition of two SCRR. The parameters of the considered structure are FS_L_ = 0.37, FS_R_ = 0.63, R_a_ + R_b_ = 50 um, C_ab_ = 0.1. When the reflection phase (R-phase) has the opposite sign, the Zak phase is 0, otherwise it is π. Therefore, we can see that the TIS is obtained at the normalized frequency Ω = 1 in this study cases.

**Figure 3:**
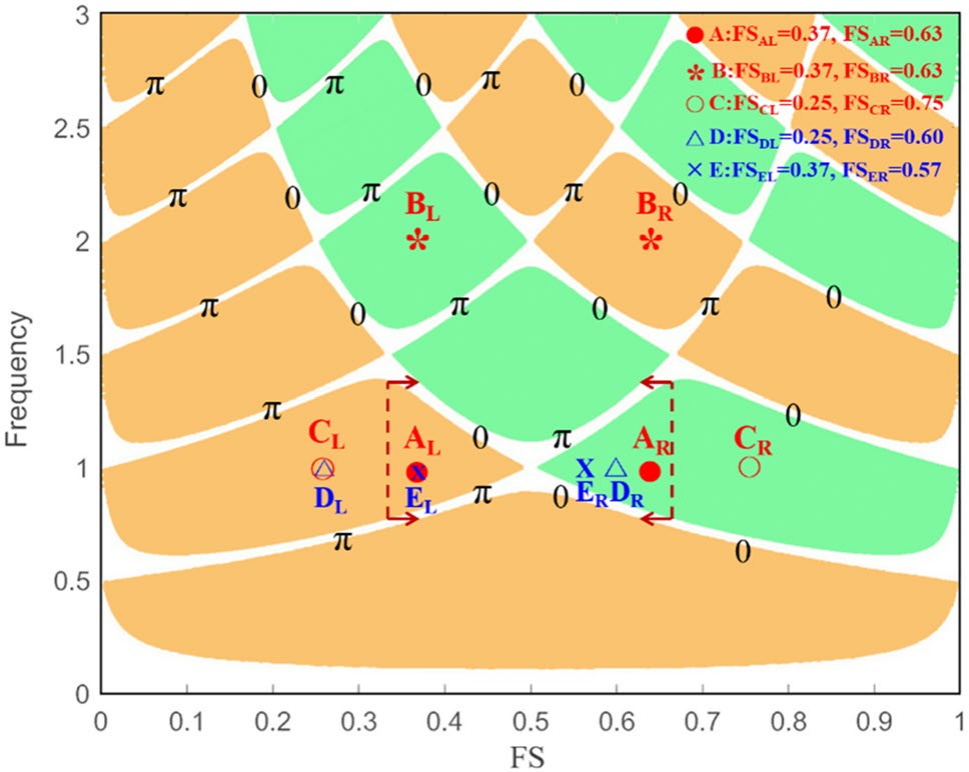
A band structure for the T-SCRR and the normalized frequency versus the filling factor of structure (FS). The parameters of the considered structure are R_a_ + R_b_ = 50 um, C_ab_ = 0.1, N = 4.

Then, we examined the band structure for the T-SCRR and the normalized frequency versus the filling factor of structure. The parameters of the considered structure are R_a_ + R_b_ = 50 um, C_ab_ = 0.1, N = 4. For clarity, only major gaps are depicted in the band structure. The orange region represents positive reflection phases, and the green region represents negative reflection phases. It was found that when the FS value is between 0.1 and 0.33, it falls in the region of CIS. Moreover, when the FS value is between 0.34 and 0.66, it falls in the region of CTIS, as shown in Fig. 3. We determined that the CTIS can be excited when the phase changes from 0 to π or from π to 0 and the reflection phase changes from positive to negative or from negative to positive. We can also clearly find the CTIS (point A), CIS (point C), IS (point D), and TIS (point E) regions at the normalized frequency Ω = 1 from the band structure. It can obtain their parameters of the normalized frequency and the fill factor of structure.

**Figure 4:**
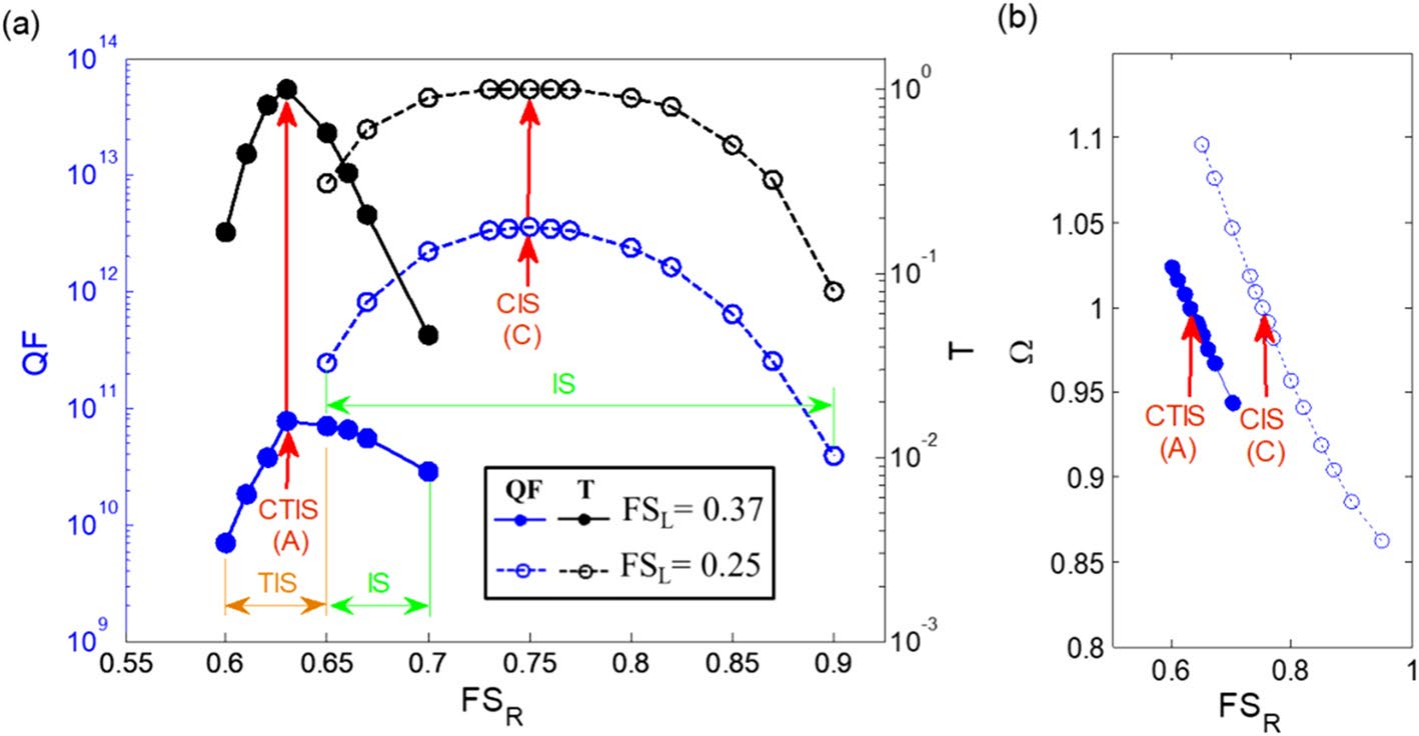
(**a**) The maximum value of quality factor (QF) and transmittance (T) versus the filling factor of FS_R_ variation for the CT-SCRR, (**b**) the normalized frequency (Ω) versus the filling factor of FSR variation for the CT-SCRR. The parameters of the considered structure are R_a_ + R_b_ = 50 um, C_ab_ = 0.1, D_1_ = D_2_.

Next, we changed the parameters and focused our attention on the two types of case studies FS_L_ = 0.25 and FS_L_ = 0.37. The parameters of the considered structure are R_a_+ R_b_ = 50 um, C_ab_ = 0.1, D_1_ = D_2_. Figure 4 shows the maximum value of the quality factor and transmittance versus the filling factor of FS_R_ variation for the CT-SCRR. The blue and black dashed curve with the hollow circle marks represents the quality factor and the transmittance versus the filling factor of FS_R_ variation for the filling factor of FS_L_ = 0.25 and the normalized frequency Ω = 1. Similarly, the blue and black solid curve with the circle marks represents the quality factor and the transmittance versus the filling factor of FS_R_ variation for the filling factor of FS_L_ = 0.37 and the normalized frequency Ω = 1, as shown in Fig. 4a. It can be observed that the filling factor of FS_R_ variation has a significant effect on the maximum quality factor and the transmittance at the normalized frequency Ω = 1. The trend curve of the maximum quality factor and the transmittance shows that it has the maximum value only in the case of conjugated states indicated by the red arrow. The CIS and the CTIS show that the two micro-ring resonators on the left and right sides are in a conjugated state to each other. The CIS indicated by the red arrow represents the maximum value of quality factor and transmittance obtained by FS_L_ = 0.25 and FS_R_ = 0.75, which are conjugated to each other. Both of these trend curves show that the quality factor and the transmittance increases rapidly to a peak reverse point, and then decreases rapidly, resembling an inverse U-like shape. These peak reversal points are always in the conjugated states. In addition, the maximum value of quality factor and transmittance are all in the IS region. A similar phenomenon occurs in the conjugated state of FS_L_ = 0.37 and FS_R_ = 0.63, as the CTIS indicated by the red arrow represents the maximum value of the quality factor and transmittance. Both of these trend lines show a sharp inverted V-shape. However, the filling factor of FS_R_ from 0.6 to 0.65 is the TIS region, and 0.65 to 0.7 is the IS region. These phenomena further prove the benefits of structural conjugation, which can produce high quality factors and perfect transmission. The normalized frequency (Ω) versus the filling factor of FS_R_ variation for the CT-SCRR is shown in Fig. 4b. We also observed that the normalized frequency changes slightly with the filling factor of FS_R_ variation.

**Figure 5:**
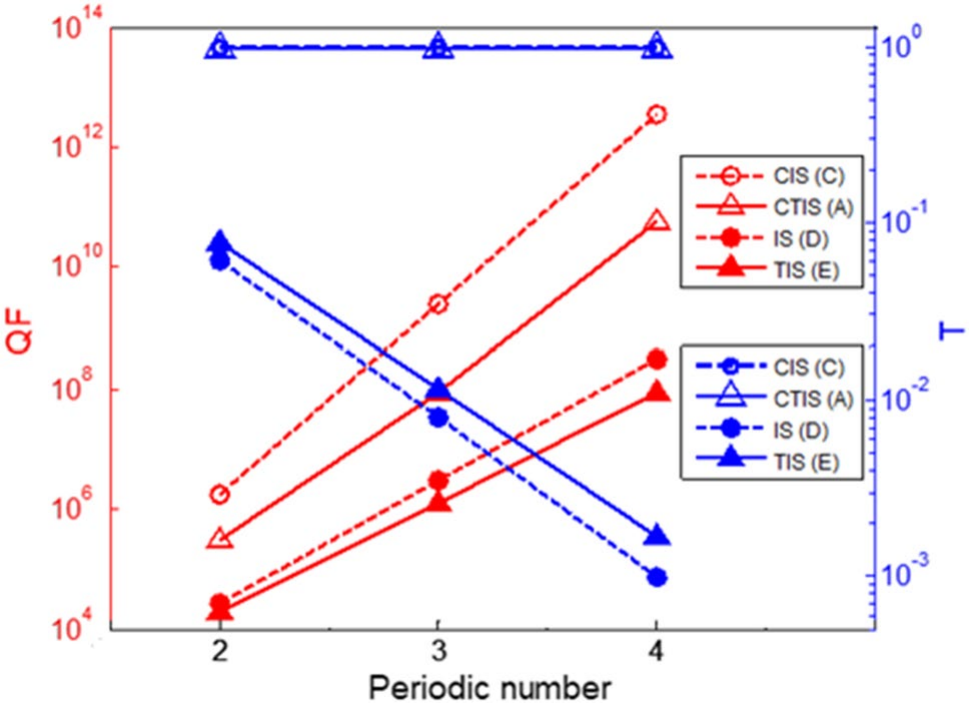
The maximum value of quality factor and the transmittance versus the periodic number variation for the T-SCRR with different types. All of the parameters are the same as those used in Fig. 2.

To clarity the optical transmission properties of the CT-SCRR, the quality factor and the transmittance versus the different periodic number is shown in Fig. 5. The red solid and dotted curves represent the quality factor for different interface states. The blue solid and dotted curves represent the transmittance for different interface states. It can be seen that the quality factor increases as the periodic number increases. We found that when the CTIS at the CT-SCRR is excited, a complete transmission is obtained and its value of transmission is equal to 1. At the same time, we also noticed that when the TIS at T-SCRR are excited, the transmission drops quickly. Furthermore, although the CIS at the SCRR has a high quality factor and complete transmission, it does not have topological protection characteristics. A brief conclusion is drawn that the CTIS has more extraordinary optical properties in these interface states. More significantly, the quality factor of the CTIS resonances increases as the periodic numbers of the CT-SCRR increases, these resonances are still perfect transmission.

**Figure 6:**
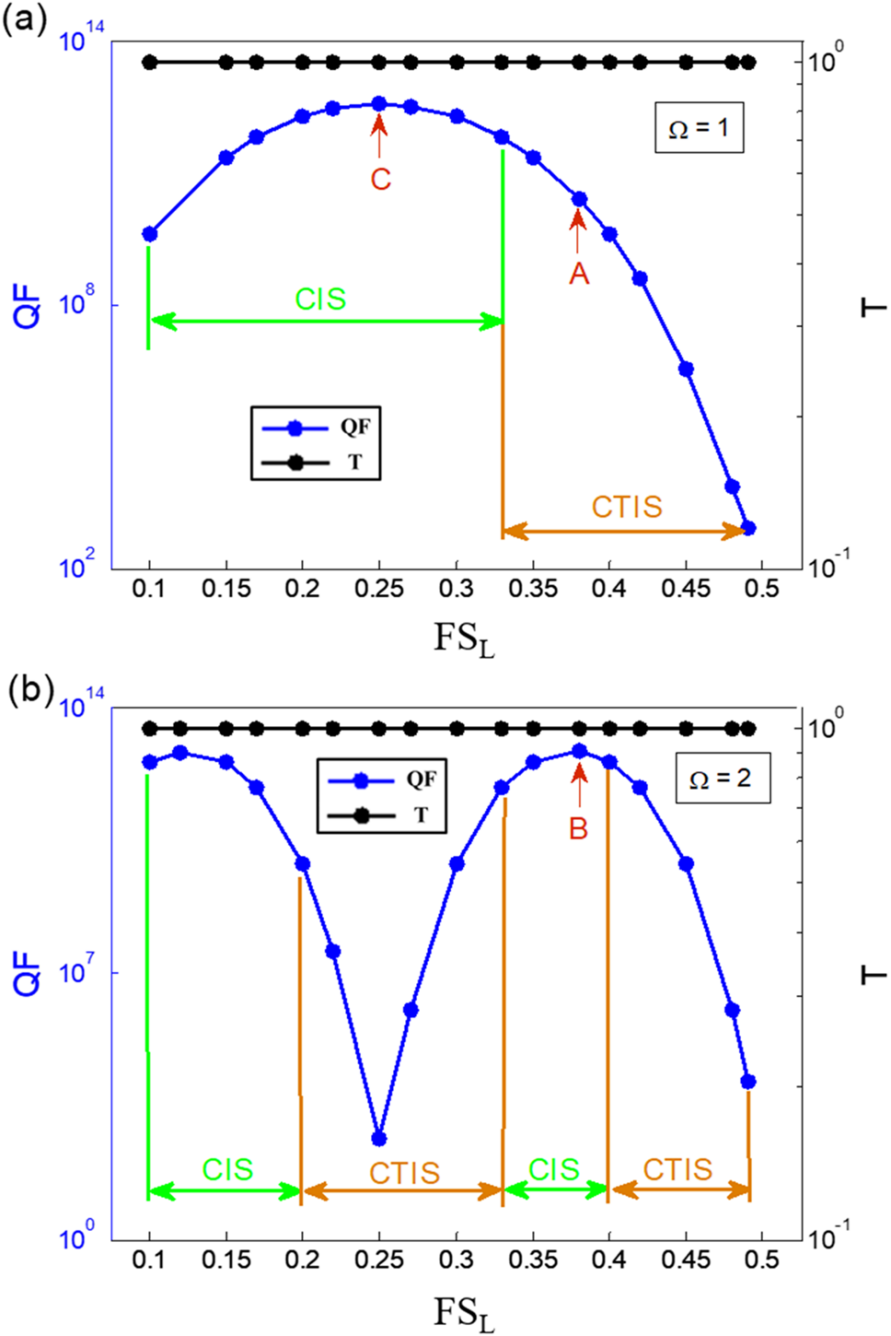
The maximum value of quality factor and transmittance versus the filling factor of FS_L_ variation for the CT-SCRR. (**a**) the normalized frequency Ω = 1, (**b**) the normalized frequency Ω = 2. The parameters of the considered structure are R_a_ + R_b_ = 50 um, C_ab_ = 0.1, N = 4, and the filling factor of FS_L_ is conjugated with FS_R_.

Finally, in order to verify the extraordinary optical characteristics of the topological interface-state in the CT-SCRR, we first distinguished the difference between the CTIS and the CIS in the considered parameters. The parameters of the considered structure are R_a_ + R_b_ = 50 um, C_ab_ = 0.1, and the filling factor of FSR is conjugated with FS_L_. Figure 6 shows the maximum value of the quality factor and transmittance versus the filling factor of FS_L_ variation for the CT-SCRR. The blue and black solid curve with the circle marks shows the quality factor and the transmittance versus the filling factor of FS_L_ variation for the normalized frequency Ω = 1, as shown in Fig. 6a. Similarly, the blue and black dashed curve with the hollow circle marks represents the quality factor and the transmittance versus the filling factor of FS_L_ variation for the normalized frequency Ω = 2, as shown in Fig. 6b. It was found that the maximum value of the quality factor in the region of CIS is much larger than the region of CTIS and has the same trend at the normalized frequencies Ω = 1 and Ω = 2. For example, in the case of the normalized frequencies Ω = 1, when the value of FS_L_ is between 0.1 and 0.33, it is in the region of CIS, the maximum value of quality factor is more of 3.58 × 10^12^ as indicated by the red arrow pointing at max. value. Moreover, when the value of FS_L_ is between 0.34 and 0.5, it is in the region of CTIS, and the maximum value of the quality factor drops rapidly. A similar phenomenon also occurs at the normalized frequencies Ω = 2. The main difference from the normalized frequency Ω = 1 is that the regional change period is shorter. The maximum value of the quality factor occurs at FS_L_ = 0.12 and FS_L_ = 0.38, and their values are 6.66 × 10^12^ and 7.14 × 10^12^ as indicated by the red arrows. It is worth noting that no matter how the parameters change, it can maintain complete transmission in the case of a conjugated structure. Therefore, we can draw a brief conclusion that the TIS and the IS both have perfect transmission in the conjugated state, but the quality factor of the IS is higher than the TIS. Moreover, the problem of transmission decreases resulting from high quality factor increases in the traditional photonic crystals^18^ is significantly improved by this approach. It is more noteworthy that even though the IS has a high-Q, it does not have topological protection.

## Conclusions

In summary, conjugated topological interface-state (CTIS) in the conjugated topological series coupled ring resonators (CT-SCRR) is first find in this article. A useful approach was successfully developed for finding the topological interface-states (TIS) in the topological series coupled ring resonators (T-SCRR). It is found that an ultra-high quality factor of more than 10^12^ with complete transmission is obtained by the CT-SCRR due to the excitation of CTIS, which is different from those in conventional topological interface-states. Moreover, even though the quality factor of the CTIS resonances increases as the periodic numbers of the CT-SCRR increases, these resonances are still perfect transmission. The problem of transmission decreases resulting from the high quality factor increases in the traditional photonic system is significantly improved by this approach. This study also improves the optical properties of the T-SCRR, which is highly susceptible to variations in manufacturing and to fluctuations in impurities. These extraordinary optical properties of the CTIS contribute to the understanding of topological CT-SCRR and open up potential applications in topological devices.

## Supplementary Information

Supplementary Information.
