# Supplementary material for: Conjugated topological interface-states in coupled ring resonators
Source: Sci Rep. 2021 Jun 8;11:12104. doi: 10.1038/s41598-021-91288-y (PMC8187389; doi:10.1038/s41598-021-91288-y)
Supplement: Supplementary file 1 — Supplementary Information. [file 41598_2021_91288_MOESM1_ESM.pdf]

Conjugated topological interface-states in coupled ring resonators

Yu-Chuan Lin[1,2], Bo-Yu Chen[1], and Wen-Jeng Hsueh[1*]

[1] Photonics Group, Department of Engineering Science, National Taiwan University, 1, Sec. 4, Roosevelt Road, Taipei, 10660, Taiwan
[2] Taiwan Instrument Research Institute, National Applied Research Laboratories, 20, R&D Rd. VI, Hsinchu Science Park, Hsinchu, 30076, Taiwan
[*] hsuehwj@ntu.edu.tw

SUPPLEMENTARY

Reflection and Zak phases

Topological series coupled ring resonators (T-SCRR) is related to the topological phase transition in the band gap. The reflection phase changes and the Zak phase changes directly govern the topological properties. In this supplementary, a T-SCRR structure was considered which consists of SCRR-L and SCRR-R as shown in the article. The SCRR-L consists of N-th half micro-rings denoted $(a'bb'a)^N$, and the SCRR-R consists of N-th half mico-rings denoted $(c'dd'c)^N$, respectively. In addition, the filling factor is a quantity which is the ratio of the radius of the two rings.

The main reason that the regions of increasing and decreasing phases asymmetric about one half filling factor is the parameter setting. In this study, the radius of ring a, b, c and d are denoted as R_a, R_b, R_c and R_d. The length of the SCRR-L is denoted as D_1, and the length of the SCRR-R is denoted as D_2, where $D_1 = R_a + R_b$, $D_2 = R_c + R_d$. The normalized frequency is denoted as $\Omega = \omega D/2\pi c$, where c is the speed of light in a vacuum. The filling factor of structure is denoted as FS, where $FS_L = R_a / D_1$, $FS_R = R_c / D_2$.

The variation in the value of FS_L and FS_R represent change in the ring dimension. In this study, we show the reflection phases and Zak phase of T-SCRR. For a one-dimensional system with inherent mirror symmetry, the Zak phase of this system takes up a quantized value of 0 or π. The relation between the sign of reflection phase and the Zak phase is given by [1-2]

$$\theta_n^{Zak} = \int_{BZ} i(\psi_k|\nabla k|\psi_k)dk,$$

where, ψ_k is the normalized Bloch eigen-function of a state with wave vector k. BZ is denoted

Brillouin zone. One-dimensional topological photonic crystals with inversion symmetry always have the Zak phase quantized at either 0 or π.

In other words, for a 1D system with inherent mirror symmetry, the Zak phase of this system takes up a quantized value of 0 or π. The relation between the sign of reflection phase and the Zak phase is given by [3-4]

$$\frac{\mathrm{sgn}(\emptyset_{n+1})}{\mathrm{sgn}(\emptyset_n)} = -\exp(i\theta_n^{Zak}), \qquad \text{with } \theta_n^{Zak} = 0 \ or \ \pi,$$

where $\emptyset_n$ is the reflection phase of the nth bandgap and θ_n^{Zak} is the Zak phase of the nth band, which is the band sandwiched by the (n+1)th and nth bandgap.

The band structures of the two T-SCRR are calculated individually by using the dispersion relation [5-6]

$$\cos(\mathrm{qA}) = \cos(k_1 d_1)\cos(k_2 d_2) - \frac{1}{2}\left(\frac{Z_1}{Z_2} + \frac{Z_2}{Z_1}\right)\sin(k_1 d_1)\sin(k_2 d_2) \ ,$$

where q is the Bloch wave vector, $k_i = \omega n_i / c,\ n_i = \sqrt{\varepsilon_i \mu_i}\ ,\ Z_i = \sqrt{\mu_i/\varepsilon_i}$

The transfer matrix method (TMM) is based on the forward and backward propagating electric fields. In this study, the forward and backward fields are included in the total transverse fields H_y and E_x. We can derive the matrix as follows [1,6].

$$\begin{bmatrix} H_y \\ E_x \end{bmatrix} = M_d \begin{bmatrix} H_y \\ E_x \end{bmatrix} = \begin{bmatrix} cosk_z d & -ik_0\varepsilon/(k_z Z_0) sink_z d \\ -ik_z Z_0/(k_0\varepsilon) sink_z d & cosk_z d \end{bmatrix} \begin{bmatrix} H_y \\ E_x \end{bmatrix},$$

where only transverse magnetic (TM) polarization is shown and to get transverse electric (TE) results one just exchange E and H.

Figure 1 (a) shows the resonance transmission spectrum of T-SCRR. The transmission peaks are obtained at the normalized frequency Ω = 1, as shown by the red arrow pointing. Figure 1 (b) and (c) show the reflection phases of SCRR-L and SCRR-R respectively. The parameters of the considered structure are FS_L = 0.25, FS_R = 0.75, $R_a + R_b$ = 50 um, C_{ab} = 0.1. When the reflection phase has the opposite sign, the Zak phase is π, otherwise it is 0. The interface states are obtained in this study cases.

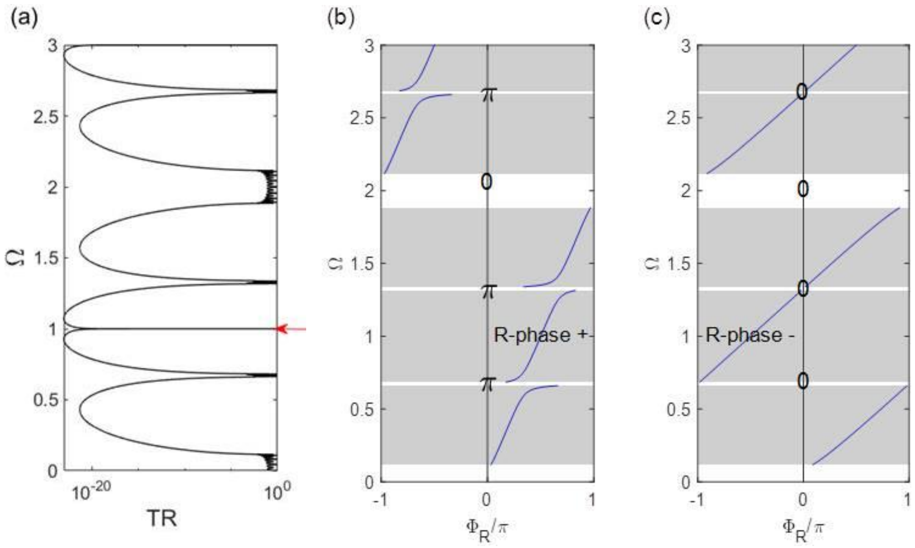

Figure 1. (a) Transmission spectrum of T-SCRR, (b) Reflection phases of SCRR-L, (c) Reflection phases of SCRR-R. The parameters of the considered structure are FS_L = 0.25, FS_R = 0.75, $R_a + R_b$ = 50 um, C_{ab} = 0.1.

Quality factor

The Q factor is a parameter that describes the resonance behavior of a resonator. The Q-factor is given by [7]

$$Q = \frac{\omega_0}{\Delta\omega} ,$$

where ω_0 is the resonant frequency, $\Delta\omega$ is the resonance width or full width at half maximum (FWHM).

The maximum value of quality factor versus the filling factor of FS_L variation for the T-SCRR with different types normalized frequency as shown in Figure 2. The red curve and green curve represent the maximum quality factor with the coupling coefficient of 0.1 and 0.2 at the same normalized frequency, Ω = 1. Similarly, the blue curve and black curve represent the maximum quality factor with the coupling coefficient of 0.1 and 0.2 at the same normalized frequency, Ω = 2. It is found that the maximum value of quality factor is obtained when the normalized frequency is 1 and the coupling coefficient is 0.1.

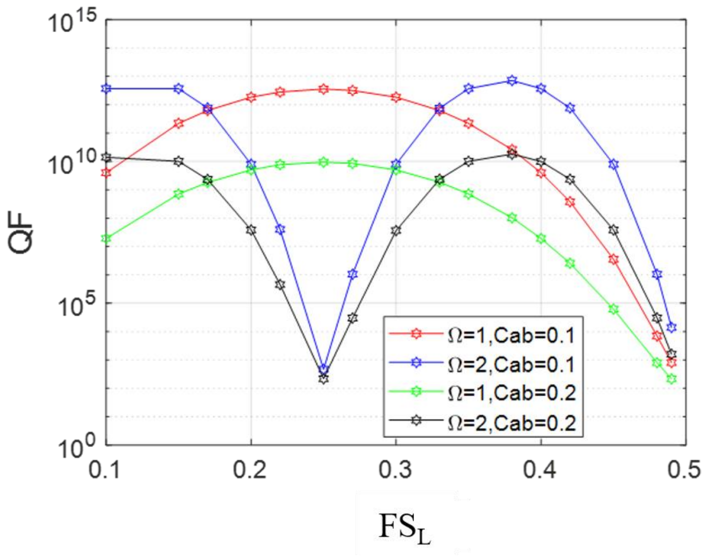

Figure 2. The maximum value of quality factor versus the filling factor of FS_L variation for the T-SCRR with different types normalized frequency.

Reference

[1] Xiao, M., Zhang, Z. Q., Chan, C. T. Surface impedance and bulk band geometric phases in one-dimensional systems. Phys. Rev. X 4, 021017 (2014).

[2] Ota, Y., Takata, K. Ozawa, T., Amo, A., Jia, Z., Kante, B., Notomi, M., Arakawa, Y. Iwamoto, S. Active topological photonics. Nanophotonics 9, 547-567 (2020).

[3] Gao, W. S., Xiao, M., Chan, C. T., Tam, W. Y. Determination of Zak phase by reflection phase in 1D photonic crystals. Opt. Lett. 40, 5259-5262 (2015).

[4] Choi, K. H., Ling, C. W., Tsang, K. F., Fung, K. H. Simultaneous multi-frequency topological edge modes between one-dimensional photonic crystals. Opt. Lett. 41, 1644-1647 (2016).

[5] Gao, H., Wei, G. G., Miao, C., Dong, P., Zhou, Y. S. Ultra-narrow unidirectional transmission filter assisted by topological interface state in one-dimensional photonic crystal heterostructure. J. Optics 48, 393–399 (2019).

[6] Guo, J., Wang, H., Dai, X., Xiang, Y., Tang, D. Enhanced nonlinear optical responses of graphene in multi-frequency topological edge modes. Opt. Express 27, 32746-32763 (2019).

[7] Christopoulos, T., Tsilipakos, O., Sinatkas, G., Kriezis, E. E. On the calculation of the quality factor in contemporary photonic resonant structures. Opt. Express 27, 14505-14522 (2019).
